# Mast cell deficiency in mice results in biomass overgrowth and delayed expulsion of the rat tapeworm *Hymenolepis diminuta*

**DOI:** 10.1042/BSR20180687

**Published:** 2018-11-30

**Authors:** Marisol I. González, Fernando Lopes, Derek M. McKay, José L. Reyes

**Affiliations:** 1 Laboratorio de Inmunología Experimental y Regulación de la Inflamación Hepato-Intestinal, UBIMED, FES Iztacala UNAM, Estado de México; 2 Institute of Parasitology, McGill University, Sainte-Anne-de-Bellevue, QC, Canada; 3 Gastrointestinal Research Group and Inflammation Research Network, Department of Physiology and Pharmacology, Calvin, Joan and Phoebe Snyder Institute for Chronic Diseases, Cumming School of Medicine, University of Calgary, Calgary, Alberta, Canada

**Keywords:** Cestode, Hymenolepis diminuta, Mast cells, Th2

## Abstract

Infection with helminth parasites evokes a complex cellular response in the host, where granulocytes (i.e. eosinophils, basophils and mast cells (MCs)) feature prominently. In addition to being used as markers of helminthic infections, MCs have been implicated in worm expulsion since animals defective in c-kit signaling, which results in diminished MC numbers, can have delayed worm expulsion. The role of MCs in the rejection of the rat tapeworm, *Hymenolepsis diminuta*, from the non-permissive mouse host is not known. MC-deficient mice display a delay in the expulsion of *H. diminuta* that is accompanied by a less intense splenic Th2 response, as determined by *in vitro* release of interleukin (IL)-4, IL-5 and IL-13 cytokines. Moreover, worms retrieved from MC-deficient mice were larger than those from wild-type (WT) mice. Assessment of gut-derived IL-25, IL-33, thymic stromal lymphopoietin revealed lower levels in uninfected MC-deficient mice compared with WT, suggesting a role for MCs in homeostatic control of these cytokines: differences in these gut cytokines between the mouse strains were not observed after infection with *H. diminuta*. Finally, mice infected with *H. diminuta* display less severe dinitrobenzene sulphonic acid (DNBS)-induced colitis, and this beneficial effect of the worm was unaltered in MC-deficient mice challenged with DNBS, as assessed by a macroscopic disease score. Thus, while MCs are not essential for rejection of *H. diminuta* from mice, their absence slows the kinetics of expulsion allowing the development of greater worm biomass prior to successful rejection of the parasitic burden.

## Introduction

Helminth parasites can persist for long periods of time inside their hosts. The effectiveness of these parasites to control host immunity is associated with their ability to trigger and maintain tissue-remodeling Th2 responses rather than inflammatory host-compromising Th1 responses [1]. In fact, this Th2-inducing ability is a helminth-intrinsic feature as evidenced by free-living worms that can condition immune cells towards a Th2 profile [2]. This canonical helminth-induced Th2 response comprises increased secretion of molecules, such as interleukin (IL)-4, IL-5 and IL-13 [3] as well as recently uncovered tissue-derived cytokines like IL-25 [4], IL-33 [5] and thymic stromal lymphopoietin (TSLP): [6] the latter three are produced early in infection, creating a local microenvironment in which T cells can fully polarize into Th2 cells. Once differentiated, Th2 cytokines perform a variety of functions aimed at expelling intestinal worms. This helminth-elicited Th2 response promotes differentiation of plasma cells and IgG_1_ and IgE production, and, in the gut, goblet cell hyperplasia, enhanced mucin secretion and accelerated peristalsis.

This Th2 cytokine response is complemented by cellular components that expand (high numbers in blood) and infiltrate organs where worm parasites dwell. Notably, this includes increased numbers of granulocytes, such as eosinophils [7], basophils [8] and mast cells [9–11]. Despite the association of these cells with infection with helminths, the precise role of each granulocyte population remains controversial, due largely to the complexity and variety of helminths examined, as well as the life cycle stage of the helminth assessed (larvae versus adults), and host genetics. However, these granulocytes are important initiators, drivers and effectors of Th2 immunity [12–14].

Mast cells (MCs) are bone marrow derived granulocytes that reside in mucosal tissues and other surfaces (i.e. skin), strategically positioned to respond to environment-derived threats. MCs play diverse roles in a variety of settings such as resistance to skin virus [15] and bacteria [16] by releasing the antimicrobial peptide cathelicidin, maintaining the intestinal epithelial barrier [17], and interacting with other myeloid cells to create a tumor-permissive environment [18]. MCs have the ability to shape the immune response through release of inflammatory cytokines (IL-17 [19] and TNFα [20]) and the Th2-promoting IL-4 [21] and IL-33 [22].

In the context of infection with helminth, early studies described anti-worm features exerted by MCs upon IgE ligation. For instance, MCs are important in the expulsion of intestinal nematodes such as *Heligmosomoides polygyrus* [22,23], *Trichuris muris* [22], *Strongyloides venezuelensis* [24], *Trichinella spiralis* [25] and *Strongyloides ratti* [26]. On the other hand, the role of MCs is controversial with *Nippostrongylus brasiliensis* [18,25]. This suggests that diversity in worm life cycle stages and host may determine the response displayed by MCs during helminth-infections and disease outcome.

Rats infected with low infective doses of the rat tapeworm, *Hymenolepis diminuta*, harbor adult worms indefinitely. The role of MCs in *H. diminuta*-infected rats has been explored. For example, F344 rats infected with five *H. diminuta* cysticercoids (never expelled) elicit a mild mast cell and IgE response [27] whereas a more prominent MC response occurs in Sprague Dawley rats infected with 35 cysticercoids, of which seldom more than ten helminths were recovered at necropsy [28]. In addition, MCs appear to have a limited role in the WsRC rat response to *H. diminuta* [29], and while a MC response was observed in infected Brown-Norway rats, there was no evidence of worm expulsion [30]. Thus, while evidence of MC activation can be found following infection with *H. diminuta*, this appears to be host- and dose-dependent, and it is far from clear the contribution, if any, of MCs to worm expulsion from the gut [31].

Mice are a highly resistant host and expel *H. diminuta* within 7–10 days of a primary infection. The mouse-*H. diminuta* model has been used to dissect the immunoregulatory circuits elicited by a small intestine-dwelling worm lacking abrasive structures that result in negligible tissue-damage [32]. While we have previously shown that MCs are activated in the mouse following infection, as evidenced by increased serum levels of mast cell protease (MCPT)-1 [33], their role in worm expulsion is not established. Here, we addressed the role of MCs in mice infected with *H. diminuta* by using animals with the *w-sh* mutation that results in suppressed expression of the *kit* molecule and consequently MC-deficiency [34].

## Materials and methods

### Mice, parasites and infection

The *H. diminuta* life cycle is maintained by infecting the flour beetle intermediate host (*Tribolium spp.*) through feeding with gravid proglotids collected from adult worms harvested from rats (Harlan laboratories, QC Canada). Five infective cysticercoids collected from infected beetles were orally gavaged in 0.9% NaCl sterile solution into wild-type (WT) and mast cell-deficient mice (Kit^w-sh^). In order to determine any differences in terms of time of expulsion, mice from both groups were killed at 8, 10, 12 and 14 days post-infection (p-i.) and parasites enumerated and worm length determined.

### Mast cell protease (MCPT)-1 quantification

Seven- to eight-week-old male WT mice and age-matched Kit^w-sh^ were orally infected with five csyticercoids of *H. diminuta* and on 8 days p-i., portions of mid-small intestine were excised and homogenized in bovine serum albumin (BSA)-containing PBS (1%) and a protease inhibitor cocktail (Roche) by using beads (50 mg/ml) in a bullet blender. Homogenate samples were diluted (1:100) and MCPT-1 measured by ELISA (eBioscience, San Diego, CA, U.S.A.).

### Cell culture

At indicated time points, spleens were aseptically removed and passed through a 100 μm mesh (Cell strainer, Falcon U.S.A.). Erythrocyte lysis was achieved by incubating the cells in ammonium chloride buffer, and leukocyte suspensions were adjusted to 3 × 10^6^/ml in RPMI-1640 medium supplemented with 10% FBS (Gibco, U.S.A.), 0.1 mM glutamine (Glutamax, Gibco, U.S.A.) and antibiotics (Pen-Strep solution, Gibco, U.S.A.). Cells were seeded for *in vitro* re-stimulation with concanavalin A (2 μg/ml) for 48 h. Following incubation, cell culture supernatants were collected and frozen (−80°C) for use in ELISA, as previously reported [35].

### Small intestine homogenates

Three centimeter segments of mid-jejunum from infected WT and Kit^w-sh^ were flushed with sterile ice-cold PBS and immediately placed in protease-containing buffer. Tissue samples were homogenized for 60 s using a tissue homogenizer (Polytron MR2100, Kinematica AG, Switzerland). After centrifugation (3000 rpm/10 min) supernatants were collected and frozen for cytokine measurements.

### ELISA sandwich

Canonical Th2 cytokine levels (i.e. IL-4, IL-10 and IL-13) were measured in splenocyte culture supernatants (3 × 10^6^ splenocytes, 2 μg/ml concanavalin A, 48-h incubation at 37°C) and tissue-derived cytokines (i.e. IL-25, IL-33 and TSLP) in jejunum homogenates. ELISAs were conducted following manufacturer’s instructions (R&D Systems Inc., Minneapolis, MN, U.S.A.).

### Flow cytometry and depletion of neutrophils

Neutrophils in spleen cell suspensions were determined by flow cytometry and phenotype was assessed by staining with APC-CD11b, PE-Gr1 and FITC-MHCII (all from Biolegend, U.S.A.), where neutrophils exhibited the phenotype SSC^+^CD11b^+^Gr1^+^MHCII^−^.

When indicated, neutrophils were depleted in Kit^w-sh^ mice by intraperitoneal (i.p.) delivery of anti-Gr1 depleting antibody (Biolegend U.S.A. Clone RB6-8C5). By using this clone, others [36–39] and we [40] have shown high specificity and effectiveness in terms of neutrophil depletion. Depleting protocol consisted of daily injections of 100 μg of anti-Gr-1 from day 0 to day 5 p-i.

### Induction and assessment of experimental colitis

Acute colitis was induced by intrarectal (ir.) delivery of 5 mg/mouse of dinitrobenzene sulfonic acid (DNBS, MP Biomedicals, Ohio, U.S.A.) in 100 μl of 50% ethanol. Weight was recorded daily for 3 days, and experimental mice were scored on a macroscopic disease activity score consisting of a 5 point scale based on weight loss, colon length, stool consistency and general appearance as previously [35].

### Statistical analysis

Data are presented as mean ± the standard error of the mean (SEM) and statistical differences were determined by one-way ANOVA followed by post-hoc analysis with Student’s *t* test or Kneuman’s Keuls test and *P*<0.05 accepted as a statistically significant difference (Graph Pad prism V5 software, La Jolla, CA, U.S.A.).

## Results

### Mast cell deficiency causes overgrowth and delayed expulsion of *H. diminuta*

**Figure 1:**
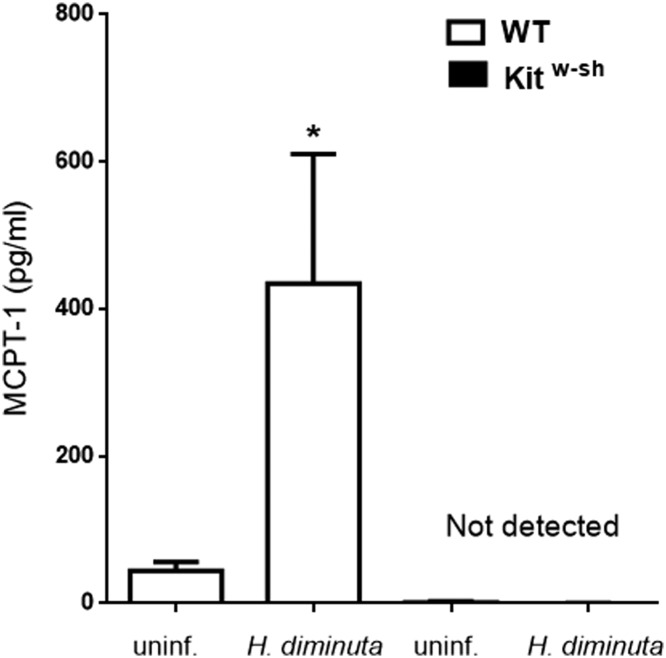
Increase in small bowel mast cell protease (MCPT)-1 following infection with *H. diminuta* To test if *H. diminuta* infection triggers MCs activation, we measured MCPT-1 levels as putative marker of such activation. Wild-type (WT) and mast cell deficient (Kit^w-sh^) mice were infected, and 8 days later jejunal tissues were excised, homogenized and MCPT-1 determined by ELISA. Data are from one experiment (*n*=4) where *P<0.05 compared against strain-matched uninfected animals.

**Figure 2:**
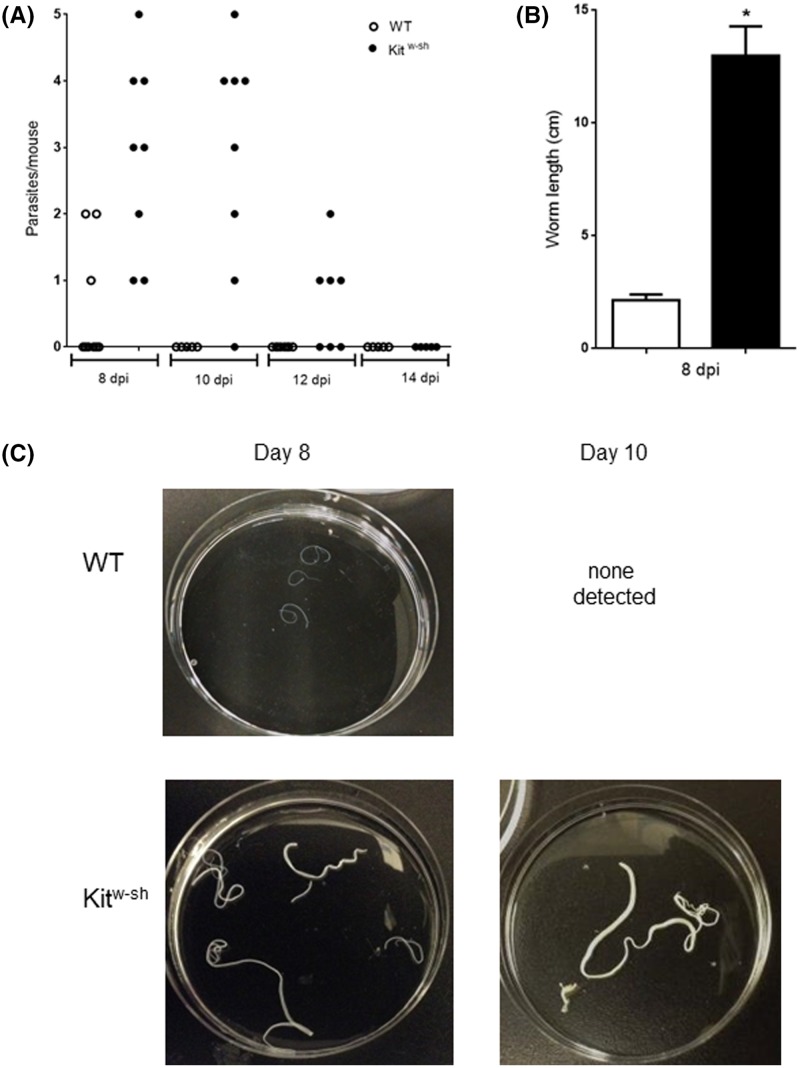
Kit^w-sh^ mice display delayed expulsion of *H. diminuta* Wild-type (WT) and mast cell-deficient (Kit^w-sh^) mice were infected with 5 cysticercoids of *H. diminuta* by oral gavage. At 8, 10, 12 and 14 days post-infection, mice under deep anesthesia were necropsied, the intestines flushed with ice-cold PBS and recovered worms counted (**A**). Parasites were measured (**B**) and representative images are shown in panel (**C**) (data are mean ± SEM; *n* = 3–8; *P<0.05 compared with time-matched WT mice).

*H. diminuta* substantially increased the levels of the MC-specific marker, MCPT-1, in small intestine of WT animals whereas samples from infected Kit^w-sh^ mice showed no increase in MCPT-1 (Figure 1). The tapeworm *H. diminuta* is expelled from the gut of immunocompetent mice within 7–10 days of a primary infection [32]. These findings were confirmed here: only one worm of a five-cysticercoid infection was retrieved on day 8 post-infection (p-i.) and none on day 10 from WT mice (Figure 2A). In contrast, Kit^w-sh^ mice showed altered kinetics of worm expulsion, harboring up to 4 worms at day 8 and 1-2 worms on day 12 (Figure 2A). Notably, in addition to delayed expulsion we observed that worms flushed from intestines of Kit^w-sh^ mice were significantly larger than those from WT mice (Figure 2B). Representative images of collected worms from both experimental groups are shown on Figure 2C. Thus, MCs assist the expulsion of *H. diminuta* worms from the mouse host and restrain increases in helminth biomass.

### Absence of MCs altered cytokine production in response to *H. diminuta* infection

**Figure 3:**
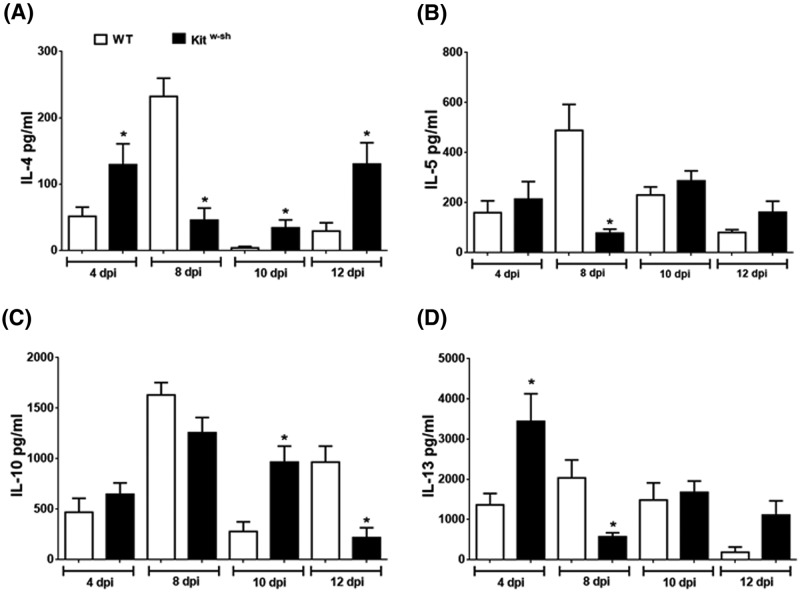
Spleen cells from infected Kit^w-sh^ mice produced an altered pattern of Th2 cytokines On the indicated days post-infection with 5 cysticercoids of *H. diminuta*, 3 × 10^6^ spleen cells were stimulated with conA (2 μg/ml) for 48 h, cell-free supernatant collected and (**A**) IL-4, (**B**) IL-5, (**C**) IL10 and (**D**) IL-13 levels determined by ELISA. Data are mean ± SEM from one experiment of three experiments yielding similar results (*n* = 3–4 each experiment) (*P<0.05 compared with time-matched WT samples).

Mice infected with *H. diminuta* display a canonical Th2 response as determined by splenocyte production of IL-4, IL-5, IL-13 and regulatory cytokine IL-10, which typically peak at ∼8 days p-i. We sought to determine the cytokine profile produced by splenocytes and compare these to those produced by mice lacking MCs. Mitogen-stimulated splenocytes from infected WT mice resulted in the expected [41] pattern of cytokine release with peak production on day 8 p-i. (Figure 3). Splenocytes from Kit^w-sh^-infected mice also displayed a Th2 response, which while not radically different from the pattern of WT mice showed distinct differences (Figure 3). Thus, the peak output of IL-4 and IL-13 occurred on day 4 p-i., there was a general reduction in IL-4, IL-5 and IL-13 on day 8 p-i. compared with WT cells and there appeared to a compensatory rebound on day 12 p-i. (Figure 3). With respect to splenic IL-10 (Figure 3C), the most notable difference between WT and Kit^w-sh^ mice was the reduced levels at day 12 p-i. in the MC-deficient mice, perhaps hinting at reduced recovery and resetting of the immune homeostatic set-point.

**Figure 4:**
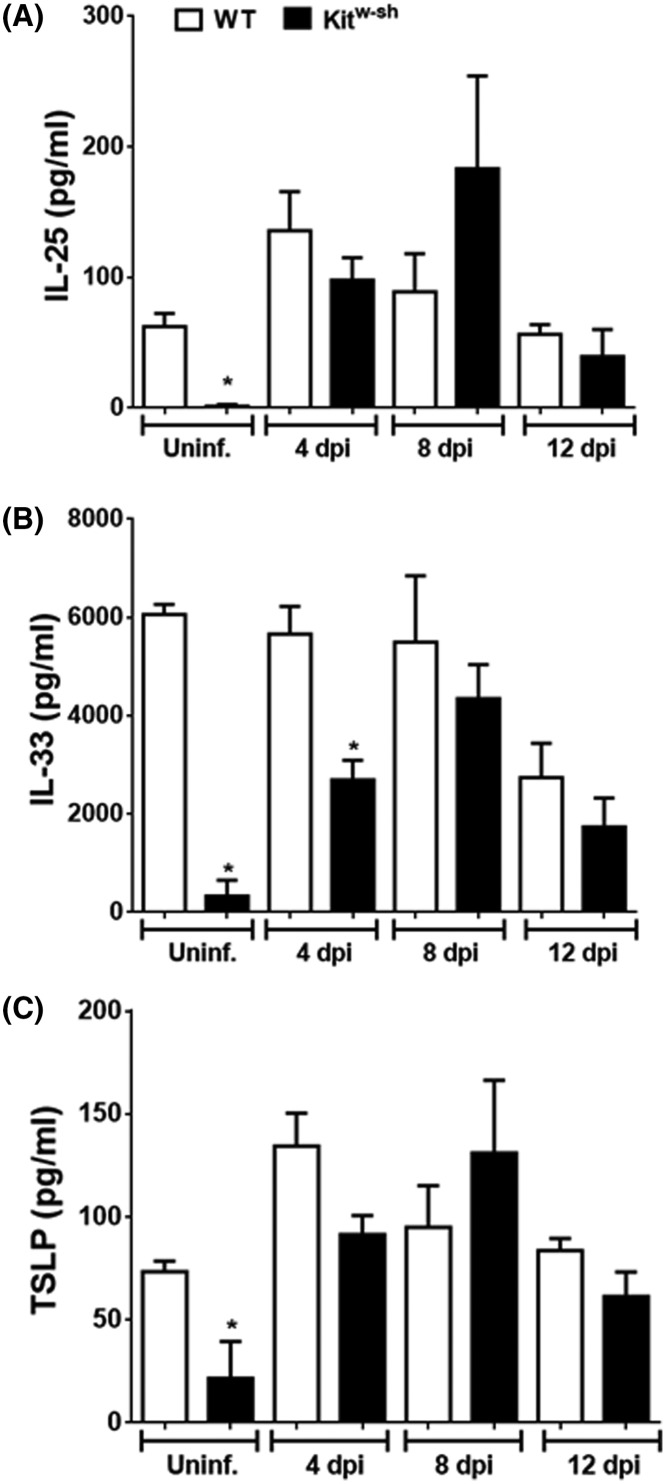
MCs regulate baseline expression of gut-derived alarmins but not those evoked by infection with *H. diminuta* On the indicated days post-infection with 5 cysticercoids of *H. diminuta*, (**A**) IL-25, (**B**) IL33 and (**C**) TSLP were measured in jejunal homogenates by ELISA. Data are mean ± SEM from one representative experiment (*n* = 3–4), two additional experiments were carried out yielding similar results (*P<0.05 compared with time-matched WT samples).

Tissue-derived cytokines prime resident cells to initiate and amplify immune responses. In the context of intestinal helminth infections, secretion of IL-25, IL-33 and TSLP promote the Th2 response [22]. We measured these cytokines in jejunal homogenates from uninfected mice as well as various time points p-i. in both experimental groups. MCs appear to be important in the basal expression of all three epithelial-derived alarmins, as jejunum from uninfected mice had very low levels compared with gut from WT mice (Figure 4). In contrast, with the exception of reduced IL-33 at day 4 p-i. in the Kit^w-sh^ gut, there were no other remarkable differences between *H. diminuta*-infected WT or MC-deficient mice (Figure 4).

### Neutrophils depletion results in accelerated expulsion of *H. diminuta*

**Figure 5:**
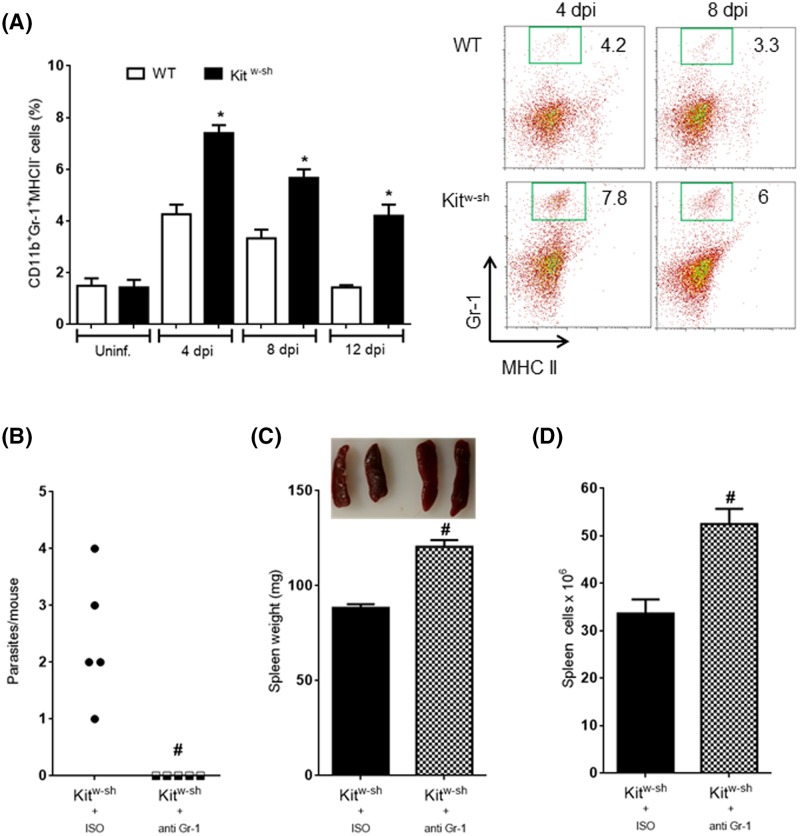
Neutrophil depletion results in splenomegaly and rapid clearance of *H. diminuta* worms Spleen cell suspensions from infected Kit^w-sh^ mast-cell deficient mice treated with either isotype control (ISO) antibodies or anti-Gr-1-depleting antibodies were adjusted to 1×10^6^/ml and incubated with blocking anti-CD16/32 and subsequently stained with fluorochrome-conjugated CD11b, Gr-1 and MHC II-specific antibodies. The percentage of neutrophils (Gr-1^+^MHCII^−^) in samples from both experimental groups at indicated time points with representative plots are shown in (**A**). Panel (**B**) shows the number of *H. diminuta* flushed from the small intestine on day 8 post-infection from Kit^w-sh^ neutrophil-depleted mice and Kit^w-sh^ mice with an intact neutrophil population. Splenic weight and cell counts from Kit^w-sh^ mice infected with *H. diminuta* and treated with neutrophil-depleting antibodies are shown in (**C**) and (**D**). Data are mean ± SEM for *n*=5 from one experiment (*P<0.05 compared to time-matched WT counterparts; #*P*<0.05 compared to time-matched ISO-treated mice).

Since Kit^w-sh^ mice harbored worms for longer periods of time but ultimately were capable of expelling the worms, we hypothesized that other cell populations could be recruited to compensate for the lack of MCs. Flow cytometry, revealed to differences between WT and Kit^w-sh^ mice in basal numbers of neutrophils (Gr-1^+^MHCII^−^), but there was a significant expansion of these cells in infected MC-deficient mice (Figure 5). Testing the hypothesis that neutrophils could participate in the coordinated response to expel *H. diminuta* in MC-deficient mice, the neutrophil depleting anti-Gr-1 antibody was administered to Kit^w-sh^-infected mice. Remarkably, this treatment of the Kit^w-sh^-infected mice resulted in complete expulsion of *H. diminuta* by 8 day p-i., while those mice given an isotype-matched antibody all had a worm burden at this time (Figure 5B). The accelerated worm expulsion was paralleled by splenomegaly (Figure 5C,D).

### Infection with *H. diminuta* protects MC-deficient mice from chemical-induced colitis

**Figure 6:**
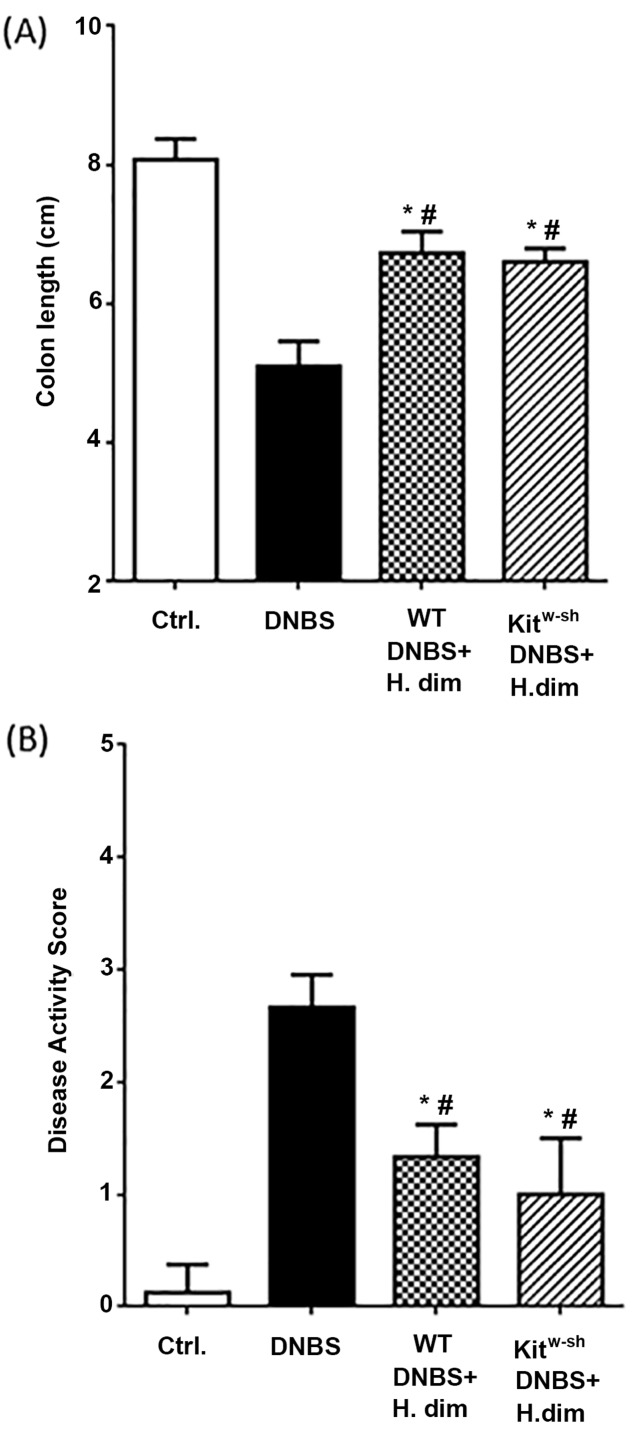
Infection with *H. diminuta* suppresses colitis in Kit^w-sh^ mice Wild-type and MC-deficient, Kit^w-sh^, mice were infected with 5 cysticercoids of *H. diminuta* (H. dim) 8 days prior to intrarectal delivery of DNBS (5 mg in 100 μl of 50% ethanol). Seventy-two hours later, colon length was measured (**A**) and a macroscopic disease score calculated (**B**). Data are mean ± SEM and *n* = 3–4 from one representative experiment of experiments (*P<0.05 as compared with control group and # compared against DNBS alone group).

We have demonstrated that infection with *H. diminuta* protects mice from DNBS-induced colitis [41]. Employing this validated model, the issue of putative mast cell involvement in the helminth-initiatied inhibition of colitis was tested, noting that the MC-deficient mice were able to eradicate the worm but with slower kinetics than WT mice (Figure 2). Interestingly, we observed comparable anti-colitic protection by *H. diminuta* infection in WT and Kit^w-sh^ mice, as gauged by colon length and macroscopic disease scores (Figure 6A,B).

## Discussion

Helminth parasites evoke Th2 responses within their hosts, which once established direct host protection [42], worm expulsion [43,44] and chronicity of the infection [45]. Of the various Th2 components, MCs are mucosa-residing cells with a rapid-response function, especially via IgE activation. In our hands, mice lacking MCs had an impaired ability to expel *H. diminuta* worms: Kit^w-sh^ mice retained parasites in the intestine until day 12 p-i., whereas worms were not detected in the gut of WT mice beyond day 10 p-i.

MC hyperplasia is a feature of infection with intestinal worm parasites; in fact, it has been shown that MCs are actively involved in the battle against helminth parasites. Studies addressing the role of MCs following worm-infections show that their importance is parasite–host specific. For example, expulsion of the nematode *T. spiralis* requires MCs, whereas that of *N. brasiliensis* does not. Pioneer studies showed that STAT6-deficient mice could not expel *N. brasiliensis* even in the presence of abundant MCs [46] and a deficiency of MCs resulted in a reduced granulomatous response in rats infected with *N. brasiliensis* [47]. In contrast, larvae *T. spiralis* remained longer in mice lacking MCPT-1, despite having abundant MCs suggesting that MCs promote *T. spiralis* expulsion [25,48]. Complementing these data, we find that MC-deficiency results in increased worm biomass and delayed expulsion of *H. diminuta* from the mouse intestine. Assessing worm biomass and infectivity in rats given 5 *H. diminuta* cysticercoids, Ohno et al. [29] reported no difference in worm biomass between WT and MC-deficient rats following primary infection, whereas upon secondary infection WT expelled a greater percentage of the worm innucolum.

While *kit* signaling is central in MC development, other signals may compensate for its absence in rats and mice. For example, MC-deficient *Ws/Ws* rats infected with *N. brasiliensis* were shown to have MCs, in fact connective tissue MCs rather than mucosal MCs are diminished in this strain [49]. Also, infection of C57BL/6 MC-deficient *w/wv* mice with the nematode *T. spiralis* resulted in the appearance of MCs [50]. Thus, while the lack of MCPT-1 in *H. diminuta*-infected Kit^w-sh^ supports the lack of MCs, the possibility that a subtype of MC arises following infection to contribute to worm expulsion cannot unequivocally be ruled out: such statement awaits an extensive investigation of mast cell markers in this mouse strain.

Given that Kit^w-sh^ mice expelled *H. diminuta* by day 14 p-i., we sought to determine if other cells were mobilized that might compensate for the absent MCs. We assessed neutrophils given their emerging putative role in the host response to infection with helminth parasites, finding them increased in spleens from infected Kit^w-sh^ mice. Many functions have been uncovered for neutrophils in helminthic infections: for example, neutrophils are central in early responses in the skin upon infection with *Litomosoides sigmodontis* [51]. Likewise, *N. brasiliensis* elicits neutrophil infiltration and NET release at the site of infection [52]. Moreover, neutrophil–macrophage cooperation has been described in the clearance of *S. stercolaris* [53] and *N. brasiliensis* [54] from mice. Importantly, neutrophil infiltration is required during the early phases of granuloma formation in response to *Schistosoma japonicum* eggs [55,56]. We observed that Kit^w-sh^ mice depleted of neutrophils presented splenomegaly and clearance of *H. diminuta*. With kinetics similar to WT mice. Others have shown that neutrophil depletion via anti-Gr-1 antibody results in an enhanced Th2 response in the context of chronic helminthic infection [57]. Further, the role of neutrophils on T-cell functions has also been reported. For instance, depleting neutrophils in the context of ocular inflammation resulted in the exacerbated Th/Th17 polarization [58], and neutrophil depletion can enhance intestinal inflammation [59]. In line with this, we speculate that neutrophil depletion enabled T cells to proliferate in a Th2-dominated microenvironment, thereby exaggerating the Th2 response resulting in accelerated expulsion of *H. diminuta*. This speculation requires testing, such as analyses of effector mechanisms associated with *H. diminuta* expulsion (e.g. goblet cell hyperplasia) in neutrophil-depleted mice. Similarly, additional studies are required to test the hypothesis that neutrophils can favor Th2 immunity following infection with helminth parasites, thus contributing to worm eradication.

The use of a model of inflammatory disease shows that infection with helminth parasites, typically in a prophylactic regimen, reduces the severity of disease [60]. Indeed, infection with *H. diminuta* has been repeatedly shown to inhibit DNBS-induced inflammation in mice, whereas B cells [61], alternatively activated macrophages [62] and IL-10 [41] have all been shown to have the potential to mediate the suppression of colitis. Given the complexity of the mucosal immune circuitry, the possibility of MC involvement in *H. diminuta*-evoked suppression of DNBS-colitis was assessed. In contrast with the kinetics of worm expulsion, Kit^w-sh^-infected mice displayed reductions in the severity of DNBS-induced colitis that was not different from that observed in *H. diminuta*-infected WT mice.

In summary, we find that MCs contribute to the rapid expulsion of *H. diminuta* as a primary infection in mice, and this is associated with nuanced changes in the ability of splenocytes to produce Th2 cytokines. In addition, two remarkable observations were made that need further investigation: first, MCs may be critical in the control of the constitutive expression of intestinal IL-25, IL-33 and TSLP; and, second, neutrophils increase in the spleen of *H. diminuta*-infected MC-deficient mice. Finally, the negligible difference in DNBS-disease severity in WT and Kit^w-sh^ mice infected with *H. diminuta* indicates that MCs are not a significant component of the *H. diminuta*-evoked anti-colitic effect.

## Abbreviations

DNBS: dinitrobenzene sulphonic acid
IL: interleukin
MC: mast cell
MCPT: mast cell protease
TSLP: thymic stromal lymphopoietin

## References

[1] Allen J.E. and Sutherland T.E. (2014) Host protective roles of type 2 immunity: parasite killing and tissue repair, flip sides of the same coin. Semin. Immunol. 26, 329–340 10.1016/j.smim.2014.06.003 25028340 PMC4179909

[2] Tawill S., Le Goff L., Ali F., Blaxter M. and Allen J.E. (2004) Both free-living and parasitic nematodes induce a characteristic Th2 response that is dependent on the presence of intact glycans. Infect. Immun. 72, 398–407 10.1128/IAI.72.1.398-407.2004 14688121 PMC343992

[3] Maizels R.M. and Yazdanbakhsh M. (2003) Immune regulation by helminth parasites: cellular and molecular mechanisms. Nat. Rev. Immunol. 3, 733–744 10.1038/nri1183 12949497

[4] Owyang A.M. (2006) Interleukin 25 regulates type 2 cytokine-dependent immunity and limits chronic inflammation in the gastrointestinal tract. J. Exp. Med. 203, 843–849 10.1084/jem.20051496 16606667 PMC1800834

[5] Humphreys N.E., Xu D., Hepworth M.R., Liew F.Y. and Grencis R.K (2008) IL-33, a potent inducer of adaptive immunity to intestinal nematodes. J. Immunol. 180, 2443–2449 10.4049/jimmunol.180.4.2443 18250453

[6] Taylor B.C. (2009) TSLP regulates intestinal immunity and inflammation in mouse models of helminth infection and colitis. J. Exp. Med. 206, 655–667 10.1084/jem.20081499 19273626 PMC2699121

[7] Strandmark J., Rausch S. and Hartmann S. (2016) Eosinophils in homeostasis and their contrasting roles during inflammation and helminth infections. Crit. Rev. Immunol. 36, 193–238 10.1615/CritRevImmunol.2016018726 28008805

[8] Eberle J.U. and Voehringer D. (2016) Role of basophils in protective immunity to parasitic infections. Semin. Immunopathol. 38, 605–613 10.1007/s00281-016-0563-3 27116557

[9] Mukai K., Tsai M., Starkl P., Marichal T. and Galli S.J. (2016) IgE and mast cells in host defense against parasites and venoms. Semin. Immunopathol. 38, 581–603 10.1007/s00281-016-0565-1 27225312 PMC5010491

[10] Basten A., Boyer M.H. and Beeson P.B. (1970) Mechanism of eosinophilia. I. Factors affecting the eosinophil response of rats to Trichinella spiralis. J. Exp. Med. 131, 1271–1287 10.1084/jem.131.6.1271 5419271 PMC2138836

[11] Pulendran B. and Artis D. (2012) New paradigms in type 2 immunity. Science 337, 431–435 10.1126/science.1221064 22837519 PMC4078898

[12] Yamanishi Y., Miyake K., Iki M., Tsutsui H. and Karasuyama H. (2017) Recent advances in understanding basophil-mediated Th2 immune responses. Immunol. Rev. 278, 237–245 10.1111/imr.12548 28658549

[13] Yasuda K. and Nakanishi K. (2018) Host responses to intestinal nematodes. Int. Immunol. 30, 93–102 10.1093/intimm/dxy002 29346656

[14] Mukai K., Tsai M., Saito H. and Galli S.J. (2018) Mast cells as sources of cytokines, chemokines, and growth factors. Immunol. Rev. 282, 121–150 10.1111/imr.12634 29431212 PMC5813811

[15] Wang Z. (2012) Skin mast cells protect mice against vaccinia virus by triggering mast cell receptor S1PR2 and releasing antimicrobial peptides. J. Immunol. 188, 345–357 10.4049/jimmunol.1101703 22140255 PMC3244574

[16] Di Nardo A., Yamasaki K., Dorschner R.A., Lai Y. and Gallo R.L. (2008) Mast cell cathelicidin antimicrobial peptide prevents invasive group A Streptococcus infection of the skin. J. Immunol. 180, 7565–7573 10.4049/jimmunol.180.11.7565 18490758 PMC2664112

[17] Groschwitz K.R. (2009) Mast cells regulate homeostatic intestinal epithelial migration and barrier function by a chymase/Mcpt4-dependent mechanism. Proc. Natl. Acad. Sci. U.S.A. 106, 22381–22386 10.1073/pnas.0906372106 20018751 PMC2799737

[18] Saleem S.J. (2012) Cutting edge: mast cells critically augment myeloid-derived suppressor cell activity. J. Immunol. 189, 511–515 10.4049/jimmunol.1200647 22706087 PMC3392490

[19] Chen X. (2015) IL-17 producing mast cells promote the expansion of myeloid-derived suppressor cells in a mouse allergy model of colorectal cancer. Oncotarget 6, 32966–32979 26429861 10.18632/oncotarget.5435 PMC4741743

[20] Gordon J.R. and Galli S.J. (1991) Release of both preformed and newly synthesized tumor necrosis factor alpha (TNF-alpha)/cachectin by mouse mast cells stimulated via the Fc epsilon RI. A mechanism for the sustained action of mast cell-derived TNF-alpha during IgE-dependent biological responses. J. Exp. Med. 174, 103–107 10.1084/jem.174.1.103 1829107 PMC2118884

[21] Brown M.A. (1987) B cell stimulatory factor-1/interleukin-4 mRNA is expressed by normal and transformed mast cells. Cell 50, 809–818 10.1016/0092-8674(87)90339-4 3497723

[22] Hepworth M.R. (2012) Mast cells orchestrate type 2 immunity to helminths through regulation of tissue-derived cytokines. Proc. Natl. Acad. Sci. U.S.A. 109, 6644–6649 10.1073/pnas.1112268109 22493240 PMC3340035

[23] Martin R.K. (2018) B1 cell IgE impedes mast cell-mediated enhancement of parasite expulsion through B2 IgE blockade. Cell Rep. 22, 1824–1834 10.1016/j.celrep.2018.01.048 29444434 PMC5832064

[24] Lantz C.S. (1998) Role for interleukin-3 in mast-cell and basophil development and in immunity to parasites. Nature 392, 90–93 10.1038/32190 9510253

[25] Knight P.A., Wright S.H., Lawrence C.E., Paterson Y.Y. and Miller H.R. (2000) Delayed expulsion of the nematode Trichinella spiralis in mice lacking the mucosal mast cell-specific granule chymase, mouse mast cell protease-1. J. Exp. Med. 192, 1849–1856 10.1084/jem.192.12.1849 11120781 PMC2213497

[26] Reitz M. (2017) Mucosal mast cells are indispensable for the timely termination of Strongyloides ratti infection. Mucosal. Immunol. 10, 481–492 10.1038/mi.2016.56 27381924

[27] Ishih A., Sekijima T., Asakawa M., Tenora F. and Uchikawa R. (2003) Hymenolepis pseudodiminuta Tenora et al. 1994 from Apodemus speciosus and H. diminuta: a comparison of experimental infections in rats. Parasitol. Res. 89, 297–301 12632167 10.1007/s00436-002-0633-4

[28] Starke W.A. and Oaks J.A. (2001) Ileal mucosal mast cell, eosinophil, and goblet cell populations during Hymenolepis diminuta infection of the rat. J. Parasitol. 87, 1222–1225 10.1645/0022-3395(2001)087%5b1222:IMMCEA%5d2.0.CO;2 11695409

[29] Ohno T. (2018) Intestinal immunity suppresses carrying capacity of rats for the model tapeworm, Hymenolepis diminuta. Parasitol. Int., 67, 357–361 10.1016/j.parint.2018.02.003 29448016

[30] Ishih A. and Uchikawa R. (2000) Immunoglobulin E and mast cell responses are related to worm biomass but not expulsion of Hymenolepis diminuta during low dose infection in rats. Parasite Immunol. 22, 561–566 10.1046/j.1365-3024.2000.00330.x 11116436

[31] Ishih A., Nishimura M. and Sano M. (1992) Differential establishment and survival of Hymenolepis diminuta in syngeneic and outbred rat strains. J. Helminthol. 66, 132–136 10.1017/S0022149X00012712 1640087

[32] McKay D.M. (2010) The immune response to and immunomodulation by Hymenolepis diminuta. Parasitology 137, 385–394 10.1017/S0031182009990886 19691904

[33] Graepel R. (2013) Murine autoimmune arthritis is exaggerated by infection with the rat tapeworm. Hymenolepis diminuta. Int. J. Parasitol. 43, 593–601 10.1016/j.ijpara.2013.02.006 23583716

[34] Tono T. (1992) c-kit Gene was not transcribed in cultured mast cells of mast cell-deficient Wsh/Wsh mice that have a normal number of erythrocytes and a normal c-kit coding region. Blood 80, 1448–1453 1381627

[35] Reyes J.L. (2016) IL-22 restrains tapeworm-mediated protection against experimental colitis via regulation of IL-25 expression. PLoS Pathog. 12, e1005481 10.1371/journal.ppat.1005481 27055194 PMC4824453

[36] Nabe T. (2011) Important role of neutrophils in the late asthmatic response in mice. Life Sci. 88, 1127–1135 10.1016/j.lfs.2011.04.003 21565205 PMC3126632

[37] Carr K.D. (2011) Specific depletion reveals a novel role for neutrophil-mediated protection in the liver during Listeria monocytogenes infection. Eur. J. Immunol. 41, 2666–2676 10.1002/eji.201041363 21660934 PMC3517125

[38] Asgharpour A., Gilchrist C., Baba D., Hamano S. and Houpt E. (2005) Resistance to intestinal Entamoeba histolytica infection is conferred by innate immunity and Gr-1+ cells. Infect. Immun. 73, 4522–4529 10.1128/IAI.73.8.4522-4529.2005 16040963 PMC1201199

[39] Tazawa H. (2003) Infiltration of neutrophils is required for acquisition of metastatic phenotype of benign murine fibrosarcoma cells: implication of inflammation-associated carcinogenesis and tumor progression. Am. J. Pathol. 163, 2221–2232 10.1016/S0002-9440(10)63580-8 14633597 PMC1892401

[40] Hunter M.M. (2010) In vitro-derived alternatively activated macrophages reduce colonic inflammation in mice. Gastroenterology 138, 1395–1405 10.1053/j.gastro.2009.12.041 20044996

[41] Hunter M.M., Wang A., Hirota C.L. and McKay D.M. (2005) Neutralizing anti-IL-10 antibody blocks the protective effect of tapeworm infection in a murine model of chemically induced colitis. J. Immunol. 174, 7368–7375 10.4049/jimmunol.174.11.7368 15905584

[42] Schwartz C., Oeser K., Prazeres da Costa C., Layland L.E. and Voehringer D. (2014) T cell-derived IL-4/IL-13 protects mice against fatal Schistosoma mansoni infection independently of basophils. J. Immunol. 193, 3590–3599 10.4049/jimmunol.1401155 25172500

[43] Anthony R.M. (2006) Memory T(H)2 cells induce alternatively activated macrophages to mediate protection against nematode parasites. Nat. Med. 12, 955–960 10.1038/nm1451 16892038 PMC1955764

[44] Finkelman F.D. (2004) Interleukin-4- and interleukin-13-mediated host protection against intestinal nematode parasites. Immunol. Rev. 201, 139–155 10.1111/j.0105-2896.2004.00192.x 15361238

[45] Reyes J.L. (2010) Early removal of alternatively activated macrophages leads to Taenia crassiceps cysticercosis clearance in vivo. Int. J. Parasitol. 40, 731–742 10.1016/j.ijpara.2009.11.014 20045000

[46] Urban J.F. Jr (1998) IL-13, IL-4Ralpha, and Stat6 are required for the expulsion of the gastrointestinal nematode parasite Nippostrongylus brasiliensis. Immunity 8, 255–264 10.1016/S1074-7613(00)80477-X 9492006

[47] Arizono N. (1996) Lung granulomatous response induced by infection with the intestinal nematode Nippostrongylus brasiliensis is suppressed in mast cell-deficient Ws/Ws rats. Clin. Exp. Immunol. 106, 55–61 10.1046/j.1365-2249.1996.d01-803.x 8870698 PMC2200549

[48] Lawrence C.E., Paterson Y.Y., Wright S.H., Knight P.A. and Miller H.R. (2004) Mouse mast cell protease-1 is required for the enteropathy induced by gastrointestinal helminth infection in the mouse. Gastroenterology 127, 155–165 10.1053/j.gastro.2004.04.004 15236182

[49] Arizono N. (1993) Infection of Nippostrongylus brasiliensis induces development of mucosal-type but not connective tissue-type mast cells in genetically mast cell-deficient Ws/Ws rats. Blood 81, 2572–2578 7683922

[50] Alizadeh H. and Murrell K.D. (1984) The intestinal mast cell response to Trichinella spiralis infection in mast cell-deficient w/wv mice. J. Parasitol. 70, 767–773 10.2307/3281760 6512640

[51] Pionnier N. (2016) Neutropenic mice provide insight into the role of skin-infiltrating neutrophils in the host protective immunity against filarial infective larvae. PLoS Negl. Trop. Dis. 10, e0004605 10.1371/journal.pntd.0004605 27111140 PMC4844152

[52] Pellefigues C. (2017) Toll-like receptor 4, but not neutrophil extracellular traps, promote IFN Type I expression to enhance Th2 responses to Nippostrongylus brasiliensis. Front. Immunol. 8, 1575 10.3389/fimmu.2017.01575 29201030 PMC5696323

[53] Bonne-Annee S. (2013) Human and mouse macrophages collaborate with neutrophils to kill larval Strongyloides stercoralis. Infect. Immun. 81, 3346–3355 10.1128/IAI.00625-13 23798541 PMC3754234

[54] Chen F. (2014) Neutrophils prime a long-lived effector macrophage phenotype that mediates accelerated helminth expulsion. Nat. Immunol. 15, 938–946 10.1038/ni.2984 25173346 PMC4479254

[55] Wu C. (2014) Schistosoma japonicum egg specific protein SjE16.7 recruits neutrophils and induces inflammatory hepatic granuloma initiation. PLoS Negl. Trop. Dis. 8, e2703 10.1371/journal.pntd.0002703 24551263 PMC3923719

[56] Chuah C. (2013) Spatial and temporal transcriptomics of Schistosoma japonicum-induced hepatic granuloma formation reveals novel roles for neutrophils. J. Leukoc. Biol. 94, 353–365 10.1189/jlb.1212653 23709687

[57] Hirata M., Hara T., Kage M., Fukuma T. and Sendo F. (2002) Neutropenia augments experimentally induced Schistosoma japonicum egg granuloma formation in CBA mice, but not in C57BL/6 mice. Parasite Immunol. 24, 479–488 10.1046/j.1365-3024.2002.00491.x 12654090

[58] Gao Y. (2015) Female-Specific downregulation of tissue polymorphonuclear neutrophils drives impaired regulatory T cell and amplified effector T cell responses in autoimmune dry eye disease. J. Immunol. 195, 3086–3099 10.4049/jimmunol.1500610 26324767 PMC4575884

[59] Kuhl A.A. (2007) Aggravation of different types of experimental colitis by depletion or adhesion blockade of neutrophils. Gastroenterology 133, 1882–1892 10.1053/j.gastro.2007.08.073 18054560

[60] Varyani F., Fleming J.O. and Maizels R.M. (2017) Helminths in the gastrointestinal tract as modulators of immunity and pathology. Am. J. Physiol. Gastrointest. Liver Physiol. 312, G537–G549 10.1152/ajpgi.00024.2017 28302598 PMC5495915

[61] Reyes J.L. (2015) Splenic B cells from Hymenolepis diminuta-infected mice ameliorate colitis independent of T cells and via cooperation with macrophages. J. Immunol. 194, 364–378 10.4049/jimmunol.1400738 25452561

[62] Johnston M.J. (2010) Extracts of the rat tapeworm, Hymenolepis diminuta, suppress macrophage activation in vitro and alleviate chemically induced colitis in mice. Infect. Immun. 78, 1364–1375 10.1128/IAI.01349-08 20028812 PMC2825920

